# Treatment success for overactive bladder with urinary urge incontinence refractory to oral antimuscarinics: a review of published evidence

**DOI:** 10.1186/1471-2490-9-18

**Published:** 2009-11-20

**Authors:** Jonathan D Campbell, Katharine S Gries, Jonathan H Watanabe, Arliene Ravelo, Roger R Dmochowski, Sean D Sullivan

**Affiliations:** 1 School of Pharmacy, University of Washington, Seattle, USA; 2 School of Pharmacy, University of Colorado Denver, Aurora, USA; 3 Global Health Outcomes Strategy & Research, Allergan, Inc. Irvine, USA; 4 Vanderbilt University Medical Center, Vanderbilt University, Nashville, USA

## Abstract

**Background:**

Treatment options for overactive bladder (OAB) with urinary urge incontinence (UUI) refractory to oral antimuscarinics include: botulinum toxin type A (BoNTA), sacral neuromodulation (SNM), and augmentation cystoplasty (AC). A standard treatment success metric that can be used in both clinical and economic evaluations of the above interventions has not emerged. Our objective was to conduct a literature review and synthesis of published measures of treatment success for OAB with UUI interventions and to identify a treatment success outcome.

**Methods:**

We performed a literature review of primary studies that used a definition of treatment success in the OAB with UUI population receiving BoNTA, SNM, or AC. The recommended success outcome was compared to generic and disease-specific health-related quality-of-life (HRQoL) measures using data from a BoNTA treatment study of neurogenic incontinent patients.

**Results:**

Across all interventions, success outcomes included: complete continence (n = 23, 44%), ≥ 50% improvement in incontinence episodes (n = 16, 31%), and subjective improvement (n = 13, 25%). We recommend the OAB with UUI treatment success outcome of ≥ 50% improvement in incontinence episodes from baseline. Using data from a neurogenic BoNTA treatment study, the average change in the Incontinence Quality of Life questionnaire was 8.8 (95% CI: -4.7, 22.3) higher for those that succeeded (N = 25) versus those that failed (N = 26). The average change in the SF-6D preference score was 0.07 (95% CI: 0.02, 0.12) higher for those that succeeded versus those that failed.

**Conclusion:**

A treatment success definition that encompasses the many components of underlying OAB with UUI symptoms is currently not practical as a consequence of difficulties in measuring urgency. The treatment success outcome of ≥ 50% improvement in incontinence episodes was associated with a clinically meaningful improvement in disease-specific HRQoL for those with neurogenic OAB with UUI. The recommended success definition is less restrictive than a measure such as complete continence but includes patients who are satisfied with treatment and experience meaningful improvement in symptoms. A standardized measure of treatment success will be useful in clinical and health economic applications.

## Background

Overactive bladder disease (OAB) is defined by the Standardization Subcommittee of the International Continence Society (ICS) as urinary urgency, with or without urinary incontinence, usually with frequency and nocturia, with no proven infection or other obvious pathology [1]. As part of the National Overactive Bladder Evaluation (NOBLE) program: 16.5% of the USA population (16% of men and 16.9% of women) over 18 years of age had symptoms consistent with OAB [2]. This translates into approximately 34 million symptomatic people in the USA alone. Of the 16.5% of the adult population with symptoms consistent with OAB, 37.2% were characterized as having OAB with urinary urge incontinence (UUI) [2]. OAB significantly impacts heath related quality of life [3] and is associated with comorbidities such as increased risk of falls and fractures, increased urinary tract and skin infections, sleep disturbances, depression, and decreased sexual health [4,5]. The economic impact of OAB is considerable given its estimated attributable cost of $12.02 billion in the USA for the year 2000 [6].

Depending on severity and etiology, initial treatment of OAB is characterized by behavioral modification (timed voiding, bladder training, pelvic floor exercises, etc) and antimuscarinic drugs. Behavioral interventions for treating OAB such as bladder training are often the first line therapy for mild symptoms of urge, frequency, and/or incontinence. Bladder training involves patient education, scheduled voiding, and positive reinforcement. Other behavioral interventions include pelvic floor exercises (PFE) and biofeedback. PFEs are thought to inhibit spontaneous bladder contractions and also increase bladder outlet resistance to result in reduced leakage and increased voiding intervals. Biofeedback is a way of notifying the patient when certain physiologic events are occurring (e.g. unstable bladder contraction or proper PFE contraction).

In a Cochrane review on bladder training, treatment outcomes consisted of patient's global observations (perception of a cure or improvement in their incontinence), quantification of symptoms (adverse events, number of incontinence episodes, number of micturitions in day and night), and quality of life (Incontinence Impact Questionnaire and SF-36) [7]. Outcomes in PFEs and biofeedback include the quantification of symptom domains, and urodynamic outcomes [8].

Most urinary incontinent patients initiate treatment with antimuscarinic therapy. Six antimuscarinic drugs are currently marketed worldwide for the treatment of OAB: oxybutynin, tolterodine, propiverine, trospium, darifenacin, and solifenacin [9]. Each product has demonstrated efficacy in treating OAB symptoms but includes such common adverse events as dry mouth, constipation, headache, and blurred vision. In a review of antimuscarinic drugs versus placebo, Nabi and colleagues found that there was a lack of consistency in the types of outcomes reported by trialists [10]. Heterogeneity in urinary incontinence and other clinical characteristics in various OAB trial patient populations may partly describe such differences. We attempted to reduce clinical characteristic heterogeneity by focusing on treatment success definitions in populations that have failed antimuscarinic therapy.

If patients fail oral drug (antimuscarinic) therapy, more invasive interventions are available. Approved implantable neurostimulator devices have been used with some success, sacral neuromodulation (SNM), but are costly [11]. Surgical options such as augmentation cystoplasty (AC), and neobladder construction, or urinary diversion are available, but are limited to those that have failed the previous mentioned techniques due to elevated risk profile and complexity of the surgeries. Although still in phase II and phase III studies, botulinum toxin type A (BoNTA) is emerging as a potential treatment option for idiopathic and neurogenic OAB with urinary urge incontinence, respectively [12].

Clinicians, payers, and other health care decision makers all have interest in determining whether or not a patient management strategy is successful. In part due to patient heterogeneity, interventions that are indicated for the management of OAB with UUI lack standardization in the measurement of treatment response or treatment success. A consistent and validated treatment success metric would allow for more efficient use of healthcare resources, facilitating the clinician and patient to more transparently weigh the risk-benefit profile of treatment alternatives. A standardized success outcome would also permit more consistent value for money assessments across and beyond OAB interventions.

The objectives of this research are to review common treatment success outcomes used in both idiopathic and neurogenic OAB with UUI, focusing on the different antimuscarinic refractory intervention classes and to recommend a measure of treatment success for clinical research and health economic applications.

## Methods

### Review Methodology

We performed a review of studies that published treatment success outcomes for OAB patients with UUI refractory to antimuscarinic therapy. A computerized search of PubMed and the grey literature including the Google search engine was conducted for studies published between 1998 and 2008. Studies were also obtained from references of published reviews and accepted studies. Studies published prior to 1998 were not included in part due to the lack of appropriate outcome development. Search terms used for retrieval of botulinum toxin studies were "botulinum A toxin overactive bladder," sacral neuromodulation were "sacral neuromodulation overactive bladder," and surgical treatment studies were "augmentation overactive bladder OR clam augmentation." Abstracts were excluded from this literature review if they were not published in English, published as a review, used botulinum toxin type B as treatment, was a dose determining or safety study, included a patient population that did not have urinary urge incontinence, was conducted in animals or in pediatrics, or conducted as a case series. In addition, we required included studies to report a measure of treatment success in terms of the proportion of the study population. Data extracted from the studies included; study design, patient characteristics, intervention, follow up time, definition of success, and proportion of participants that succeeded. In our analysis we summarized by definition of treatment success, the proportion of patients that succeeded the intervention of study.

### Treatment Success Association

We selected the best candidate for a treatment success definition based in part on its emphasis and use as reported in the literature. We associated this candidate definition using patient level data from a clinical study of 59 patients with neurogenic urinary incontinence [13]. We investigated the relationship between the identified/recommended treatment success measure and both generic and disease-specific health-related quality of life (HRQoL) outcomes. We used the Incontinence Quality of Life Questionnaire (I-QOL) [14] as an estimate of the disease-specific HRQoL and preference scores from the SF-6D as an estimate of generic HRQoL. We followed the methods outlined by Brazier et al to convert existing SF-36 data into preference scores (range 0-1) [15]. The I-QOL is an incontinence-specific measure that has shown high levels of validity and reliability in stress incontinence, overactive bladder and neurogenic detrusor overactivity [14,16]. The I-QOL consists of 22 items that fall within three domains: avoidance and limiting behavior; psychosocial impacts; and social embarrassment. For each item, subjects assign a value on a 5-point Likert scale from 1 (extremely) to 5 (not at all). The total scale score for all 22-items is transformed to a 0 (extreme incontinence problems) to 100 (no incontinence problems) range.

We performed two-sample t-tests assuming equal variances to estimate the mean difference in the I-QOL and SF-6D change scores for treatment success versus treatment failure patients. The minimal important difference (MID) observed for the I-QOL instrument was a change in the total score of at least 6.3 for patients with stress urinary incontinence [17]. We used the MID as a benchmark to determine if the average difference observed in the I-QOL was clinically meaningful. Although the Schurch et al. study intervention was BoNTA compared to placebo, we did not adjust for treatment assignment or any other baseline characteristics in the validation of the treatment success outcome.

## Results

### Botulinum Toxin Type A (BoNTA)

There is an emerging body of evidence in support of the efficacy and safety of using BoNTA in OAB [12]. A review by Duthie and colleagues of randomized or quasi-randomized controlled trials of BoNTA found similar outcomes reported as in the antimuscarinic therapy reviews [18]. Changes in incontinence rates were the most frequently reported efficacy outcome followed by urinary frequency, health-related quality of life, and urodynamic measures of cystometric capacity and detrusor pressure. Schurch summarized BoNTA published studies including those with non-randomized designs for the neurogenic and idiopathic OAB populations [19]. One result from Schurch's review was a summary of the study specific success rates stratified by the neurogenic and idiopathic populations. Success in this instance was broadly based on the proportion of patients who met a threshold of reduced incontinence episodes.

**Figure 1:**
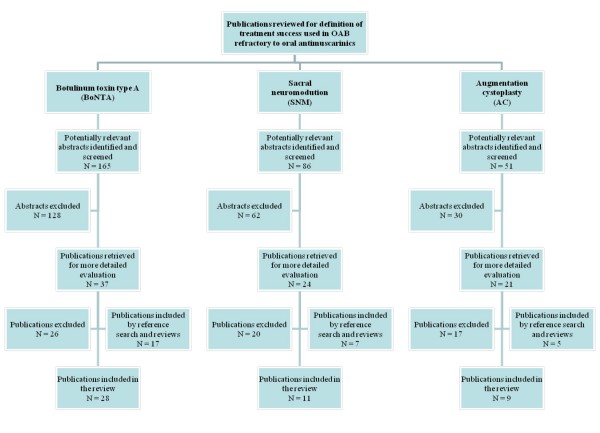
**Study search yield by intervention**.

The search of BoNTA studies yielded 165 abstracts for review (Figure 1). A total of 11 articles met inclusion criteria and an additional 17 articles were retrieved from published reviews or references of accepted articles.

### Sacral Neuromodulation (SNM)

Brazzelli and colleagues provided a review of SNM that includes a discussion of outcomes used to demonstrate the efficacy and safety as well as the success of the device [20]. A more unified definition of success has been reported within the SNM literature. Namely, the term "cured" was equated to at least a 90% reduction in incontinence symptoms and "improved" meant greater than a 50% reduction in incontinence symptoms. Together, a "cured" or "improved" patient was thought to have succeeded on SNM. Other outcome measures such as pad usage, incontinence severity, voiding frequency, urodynamic parameters, and health-related quality of life were reported to secondarily demonstrate efficacy, but not with the same consistency as the cured or improved rates. Safety outcomes mainly focused on adverse events such as reoperations, replacement or relocation of the device, permanent explants, generator or electrode/lead failures, pain, infection, wound problems, and adverse bowl function.

The search of SNM studies produced 86 abstracts for review (Figure 1). From these, 4 articles were included and 7 studies came from references of review articles.

### Augmentation Cystoplasty Surgery (AC)

Augmentation cystoplasty (AC) is a surgery where a segment of the bowel is removed and patched to the bisected bladder. This method increases bladder capacity and decreases bladder pressure caused by unstable contractions. Risks of this surgery include kidney or bladder infections, new recurrent UTIs, metabolic derangements, mucus production, and in rare cases tumors within the bladder. Khastgir et al. described outcomes measured for augmentation cystoplasty [21]. The emphasized outcomes were clinical outcomes (maximum detrusor pressure and bladder volume capacity) and the quantification of patient symptoms (incontinence episodes and number of pads). Other outcomes included a questionnaire that measured symptoms and disease specific quality of life, and the evaluation of complications from the surgery. An alternative to augmentation cystoplasty is detrusor myectomy where an excision of the detrusor muscle from the top of the bladder results in a bulge in the epithelium and in turn increases storage capacity of the bladder. Although this procedure is less risky, it is also thought to be less effective. Similar outcomes emphasizing the clinical and symptoms domains have been measured for detrusor myectomy [22].

The search of AC studies produced 51 abstracts for review (Figure 1). A total of 4 studies were included from the search, 3 studies from references of review articles, and 2 studies from references of other accepted studies.

### Summary overall interventions

Across BoNTA, SNM, and AC for OAB with UUI, 48 studies met the entry criteria for the review of treatment success definitions (58.3% BoNTA, 22.9% SNM, and 18.8% AC). Due to a small percentage of studies reporting more than one definition of treatment success, the total number of treatment success definitions was 52. Achieving complete continence was reported as a definition of treatment success 23 times (44%) (Table 1). Achieving at least a ≥ 50% reduction in incontinence episodes (or other symptoms) was reported as a definition of treatment success 16 times (31%) (Table 2). Subjective patient satisfaction measures were reported as a definition of treatment success 13 times (25%) (Table 3). Achieving at least a ≥ 50% reduction in incontinence episodes (or other symptoms) was the most common definition for the idiopathic population (42%) whereas complete continence was the most common definition for the neurogenic population (59%). We now present supporting evidence for the ≥ 50% reduction in incontinence episodes as a measure of treatment success.

**Table 1:** Complete Continence: Treatment Success Definition by Intervention

Study	Study Design	Sample Size	Study Population	Comparator	Success Evaluated (months)	Success %
**Botulinum toxin type A**						
Kennelly, Top Spinal Cord Inj Rehabil, 2003 [26]	Open Label	10	NOAB	None	6	80
Reitz, Eur Urol, 2004 [27]	Open Label	200	NOAB	None	4	73
Giannantoni, J Urol, 2004 [28]	RCT	25	NOAB	Intravesically Resiniferation	18	73
Giannantoni, Minerva Urol Nephrol, 2004 [29]	RCT	12	NOAB	Intravesically Resiniferation	12	75.0
Klaphajone, Arch Phys Med Rehabil, 2005 [30]	Open Label	10	NOAB	None	1.5	70
Schurch, J Urol, 2005 [13]	RCT	38	NOAB	Placebo	6	63
Kessler, Neurourol Urodynam, 2005* [31]	Open Label	11, 11	NOAB, IOAB	None	3, 3	72, 91
Popat, J Urol, 2005* [32]	Open Label	31, 44	NOAB, IOAB	None	4, 4	55.2, 57
Werner, Am J Obstet Gynecol, 2005 [33]	Open Label	26	IOAB	None	9	65
Kuo, J Urol, 2006* [34]	Single Blind	35, 40	NOAB, IOAB	None	3, 3	94, 73
Giannantoni, J Urol, 2006 [35]	Open Label	23	NOAB	None	3	78
Karsenty, Urol, 2006 [36]	Open Label	17	NOAB	None	5	100
Sahai, J Urol, 2007 [37]	RCT	16	IOAB	Placebo	3	50
Mascarenhas, Neurourol. Urodynam, 2008 [38]	Open label	21	NOAB	None	2	42.8

**Sacral neuromodulation**						
Bosch, J Urol, 2000 [39]	Open Label	6	NOAB	None	47	80
Chartier-Kastler, J Urol, 2000 [40]	Open Label	9	NOAB	None	43.6	67
Weil, Eur Urol, 2000 [41]	RCT	21	IOAB	Current Management	6	56
Spinelli, J Urol, 2001 [42]	Open Label	196	NOAB	None	18	71

**Augmentation cystoplasty**						
Chartier-Kaslter, Spinal Cord, 2000 [43]	Open Label	17	NOAB	None	75.6	70.5
Ivil, Int Urol Nephrol, 2002 [44]	Open Label	17	IOAB	None	11	83
Khastgir, Eur Urol, 2003 [21]	Open Label	32	NOAB	None	72	100
Quek, J Urol, 2003 [45]	Open Label	26	NOAB	None	96	69
Stoffel, Neurourol Urodynam, 2006 [46]	Open Label	12	NOAB	None	20	88

* Denotes a study with both neurogenic OAB (NOAB) and idiopathic OAB (IOAB) populations.

Note: Studies may be listed in more than one category of treatment success if multiple definitions of treatment success were reported.

**Table 2:** ≥ 50% Improvement in Incontinence Episodes or Other Symptoms: Treatment Success Definition by Intervention

Study	Study Design	Sample Size	Study Population	Comparator	Success Evaluated (months)	Success %
**Botulinum toxin type A**						
Flynn, J Urol, 2004 [47]	Open Label	7	IOAB	None	3	100
Kuo, Urology, 2004* [48]	Open Label	12, 8	NOAB, IOAB	None	3, 3	66.6, 75
Kuo, Urology, 2005 [49]	Open Label	20	IOAB	None	3	85
Kalsi, Eur Urol, 2006* [50]	Open Label	63, 38	NOAB, IOAB	None	4, 4	86, 79
Kalsi, Ann Neurol, 2007 [51]	Open Label	43	NOAB	None	4	80
Mascarenhas, Neurourol. Urodynam, 2008 [38]	Open label	21	NOAB	None	2	52.4

**Sacral neuromodulation**						
Schmidt, J Urol, 1999 [52]	RCT	34	IOAB	Current Management	6	75
Bosch, J Urol, 2000 [39]	Open Label	40	IOAB	None	47	60
Amundsen, Am J Obstet Gynecol, 2002 [53]	Open Label	12	IOAB	None	7	100
Bosch, J Urol, 2000 [39]	Open Label	6	NOAB	None	47	100
Hassouna, J Urol, 2000 [54]	RCT	25	IOAB	Current Management	6	56
Scheepens, Eur Urol, 2003 [55]	Open Label	34	IOAB	None	11	53
Van Voskuilen, BJU Int, 2007 [56]	Open Label	31	IOAB	None	15.5	90
Groenendijk, BJU Int, 2008 [57]	Open Label	67	IOAB	None	6	61
Wallace, Am J Obstet Gynecol, 2007 [58]	Open Label	33	NOAB	None	12.5	84.8

**Augmentation cystoplasty**						
Blaivas, J Urol, 2005 [59]	Open Label	76	NOAB	None	106.8	97

* Denotes a study with both neurogenic OAB (NOAB) and idiopathic OAB (IOAB) populations.

Note: Studies may be listed in more than one category of treatment success if multiple definitions of treatment success were reported.

**Table 3:** Subjective Improvement: Treatment Success Definition by Intervention

Study	Study Design	Sample Size	Study Population	Comparator	Success Evaluated (months)	Success %
**Botulinum toxin type A**						
Loch, Eur Urol Supp, 2003* [60]	Open Label	30, 30	NOAB, IOAB	None	8, 8	67, 67
Rapp, Urology, 2004 [61]	Open Label	35	IOAB	None	6	60
Grosse, Eur Urol, 2005 [62]	Open Label	66	NOAB	None	10	86.3
Rajkumar, BJU Int, 2005 [63]	Open Label	15	IOAB	None	1.5	93
Schulte-Baukloh, Eur Urol, 2005 [64]	Open Label	44	IOAB	None	3	86
Schmid, J Urol, 2006 [65]	Open Label	100	IOAB	None	3	88
Schulte-Baukloh, Neur Urodyn, 2006 [66]	Open Label	16	NOAB	None	6	100
Kuo, J Urol, 2007 [67]	Open Label	45	IOAB	None	3	80

**Augmentation cystoplasty**						
Edlund, Scand J Urol Nephrol, 2001 [68]	Open Label	25	IOAB	None	60	78
Barrington, Int Urogynecol J, 2006 [69]	Open Label	12	IOAB	None	12	83
Chartier-Kaslter, Spinal Cord, 2000 [43]	Open Label	17	NOAB	None	75.6	88.5
Quek, J Urol, 2003 [45]	Open Label	26	NOAB	None	96	96
Nomura, Spinal Cord, 2002 [70]	Open Label	11	NOAB	None	66	100

* Denotes a study with both neurogenic OAB (NOAB) and idiopathic OAB (IOAB) populations.

Note: Studies may be listed in more than one category of treatment success if multiple definitions of treatment success were reported.

### Treatment Success Association: ≥ 50% reduction in incontinence episodes

Of the 51 patients reporting incontinence scores at both baseline and at six month visits across all treatment groups in the Schurch et al trial, 25 (49%) achieved the ≥ 50% reduction in incontinence episodes definition of response. Patients with ≥ 50% reduction in incontinence episodes on average had 8.8 higher I-QOL scores (95% CI: -4.7, 22.3) and 0.07 higher SF-6D preference scores (95% CI: 0.02, 0.12).

## Discussion

Deriving a definition of treatment success in any disease area poses many challenges, but we argue that the benefits outweigh the harms in undergoing such an exercise in UUI OAB. Possible limitations to a definition of treatment success in UUI OAB include: patient clinical heterogeneity (e.g. UUI OAB patients have varying levels of incontinence), patient preference heterogeneity (e.g. some patients having the same study outcomes may opt to continue treatment whereas others may opt for alternative interventions), and duration of treatment effect (e.g. if incontinence or other symptoms are included in the measure of success, then how often and over what time period should they be measured?). At the population level, a uniform treatment response outcome provides for the comparison across various UUI OAB interventions, gives researchers a clear and achievable target for purposes of trial design, and allows payers to attain better value for money on UUI OAB interventions for their insured population.

Payne and Kelleher recently evaluated three possible treatment response definitions that were informed from the ICS definition of OAB: (i) a reduction by half or more in all baseline symptoms (episodes of urgency, incontinence, nocturia, and voiding frequency per 24 h); (ii) a reduction by half or more in urgency and at least one other symptom; or (iii) resolution of urgency episodes and at least one other symptom [23]. All three treatment response definitions were correlated with significant changes in the King's Health Questionnaire. The authors argued that clinical trials and the evidence base in OAB focus on single endpoints such as incontinence episodes or reduction in incontinence, but correctly point out that the ICS definition of OAB mainly targets urgency and secondarily lists the other symptoms of incontinence, nocturia, and increased voiding frequency. One of the main pitfalls in using any of these three proposed treatment response definitions is that there is yet to be a consensus on how to measure urgency in a reliable and valid way [24]. Further, if one is tasked to gather the current and existing evidence across OAB interventions on treatment response, then retrospectively constructing a composite definition of success is nearly impossible.

Taking into account the currently available evidence, the most likely candidates for a definition of OAB with UUI treatment success are measures that focus on reductions in incontinence episodes. The achievement of full continence has been a common metric in BoNTA studies. But in the case of a dichotomous outcome of success (yes/no), full continence likely excludes many patients that are clinically benefiting from treatment. The question then becomes, what is an appropriate threshold in terms of incontinence reduction that resonates as a clinically meaningful improvement yet does not disregard too many patients that are benefiting? We sought out a definition of success that limits misclassification/residual confounding by setting a threshold that most appropriately includes those receiving meaningful clinical benefit while excluding those that experience a measurable benefit that is not clinically relevant.

We recommend that the appropriate threshold for defining OAB with UUI treatment success should be ≥ 50% reduction in incontinence episodes from baseline. We stress that the use of this definition may be sensitive to the baseline incontinence rate and therefore, both the baseline incontinence rate and the change in the rate should be communicated in research findings. In part, the recommendation definition is based on what is currently available and reported in the literature. The ≥ 50% reduction in incontinence episodes treatment response definition has been used across all reviewed intervention classes and is associated with generic and disease specific HRQoL measures. The average difference in the disease specific HRQoL instrument, I-QOL, change scores was higher than the minimal important difference (MID) reported for stress urinary incontinence of 6.3 [17]. Although stress urinary incontinence is not the study population, it is the only known population wherein a MID for the I-QOL has been reported. Additionally, Yalcin et al reported that stress urinary incontinence patients appear to recognize important clinical value at incontinence reductions of approximately 50% [25]. If the MID for OAB with UUI was found to be similar to that in stress urinary incontinence, then the findings from the Schurch et al trial suggest that the recommended treatment success definition is associated with a meaningful improvement in a disease specific HRQoL instrument.

There are several limitations to the study. The primary aim of this research was to review the evidence on treatment success definitions reported for OAB with UUI refractory to oral antimuscarinics. We focused on three primary intervention classes in order to make our literature search tractable. By doing so, we did not summarize treatment success definitions for more rare interventions in this population such as percutaneous tibial nerve stimulation given the limited quantity of published evidence on such interventions. We did not include behavior modification interventions or antimuscarinics because of the anticipated large amount of patient characteristic heterogeneity in this broad population. Increased heterogeneity in UUI symptoms of a population could limit the generalizability of a uniform treatment success definition. The association of the recommended treatment success definition with disease specific and generic HRQoL measures was performed in a BoNTA compared to placebo trial of neurogenic incontinent patients. This same association may not hold for open label studies or those with SNM or AC interventions. Although the recommended treatment success definition of ≥ 50% reduction in incontinence episodes from baseline was the most common observed definition in the idiopathic OAB with UUI population, it should be further validated against other OAB outcomes.

## Conclusion

We echo arguments put forth by Payne and Kelleher on the need for a standardized treatment success definition. Moving forward, a success definition like those proposed by Payne and Kelleher that includes many components of the underlying symptoms of OAB (namely urgency) makes clinical sense, but is currently not practical based on difficulties in measuring urgency. Based on the current evidence, we have proposed a definition of success for OAB with UUI that is currently gaining momentum in the literature and is supported by associations with clinically meaningful outcomes. The adoption of the ≥ 50% reduction in incontinence episodes as the treatment response definition for UUI OAB will provide for consistent and valuable outcomes research for both clinical and health economic applications.

## Competing interests

JDC, RRD, and SDS are consultants to Allergan, Inc. JDC and SDS were compensated for their contributions to this manuscript through an unrestricted grant by Allergan, Inc. KSG and JHW are University of Washington fellows funded by Allergan, Inc. AR was a former employee of Allergan, Inc. The authors declare that they have no other competing interests to disclose.

## Authors' contributions

SDS, JDC, and AR, designed and specified the research objectives. KSG and JDC carried out the literature searches and data extraction from the literature. JHW and RRD were responsible for the clinical interpretation of the findings. JDC drafted the manuscript. All coauthors were responsible for critically revising the manuscript. All coauthors have given final approval of this version for publication.

## Pre-publication history

The pre-publication history for this paper can be accessed here:

http://www.biomedcentral.com/1471-2490/9/18/prepub
